# Risk factors for reported obstetric complications and near misses in rural northwest Bangladesh: analysis from a prospective cohort study

**DOI:** 10.1186/1471-2393-14-347

**Published:** 2014-10-04

**Authors:** Shegufta S Sikder, Alain B Labrique, Abu A Shamim, Hasmot Ali, Sucheta Mehra, Lee Wu, Saijuddin Shaikh, Keith P West, Parul Christian

**Affiliations:** Department of International Health, Johns Hopkins Bloomberg School of Public Health, Baltimore, MD USA; The JiVitA Maternal and Child Health Research Project, Gaibandha, Bangladesh

## Abstract

**Background:**

In rural Bangladesh, more than 75% of all births occur at home in the absence of skilled birth attendants. Population-based data are lacking on the burden and risk factors for obstetric complications in settings with low rates of institutional delivery. We sought to describe the prevalence of reported complications and to analyze risk factors for obstetric complications and near misses, using data from a representative, rural setting of Bangladesh.

**Methods:**

This study utilized existing data on 42,214 pregnant women enrolled in a micronutrient supplementation cohort trial between 2007 and 2011 in rural northwest Bangladesh. Based on self-report of complications, women were categorized as having obstetric complications, near misses, or non-complicated pregnancies using definitions modified from the World Health Organization. Multivariable multinomial regression was used to analyze the association of biological, socioeconomic, and psychosocial variables with obstetric complications or near misses.

**Results:**

Of enrolled women, 25% (n = 10,380) were classified as having at least one obstetric complication, 2% (n = 1,004) with reported near misses, and 73% (n = 30,830) with non-complicated pregnancies. Twelve percent (n = 5,232) reported hemorrhage and 8% (n = 3,259) reported sepsis. Of the 27,241 women with live births or stillbirths, 11% (n = 2,950) reported obstructed labor and 1% (n = 328) reported eclampsia. Biological risk factors including women’s age less than 18 years (Relative Risk Ratio [RRR] 1.26 95%CI:1.14-1.39) and greater than 35 years (RRR 1.23 95%CI:1.09-1.38), history of stillbirth or miscarriage (RRR 1.15 95%CI:1.07-1.22), and nulliparity (RRR 1.16 95%CI:1.02-1.29) significantly increased the risk of obstetric complications. Neither partner wanting the pregnancy increased the risk of obstetric complications (RRR 1.33 95%CI:1.20-1.46). Mid-upper arm circumference <21.5 cm increased the risk of hemorrhage and sepsis.

**Conclusions:**

These analyses indicate a high burden of obstetric morbidity. Maternal age, nulliparity, a history of miscarriage or stillbirth, and lack of pregnancy wantedness were associated with increased risk of obstetric complications. Policies to address early marriage, unmet need for contraception, and maternal undernutrition may help mitigate this morbidity burden in rural Bangladesh.

## Background

In June 2012, a series of reviews highlighted the “neglected role of morbidity in the maternal health agenda” [1–4]. While studies have focused for decades on quantifying determinants of maternal deaths, this data has “not been matched by in-depth efforts to characterize and understand the burden of obstetric complications suffered by childbearing women in developing countries” [2]. In rural Bangladesh, approximately 75% of women give birth at home in the absence of skilled birth attendants [5]. As a consequence, the majority of obstetric complications (defined as acute conditions such as sepsis, eclampsia, hemorrhage, and obstructed labor that can cause maternal deaths [1]) arise in the home. In these contexts, hospital-based studies are likely non-representative as women who deliver in hospitals are typically younger, wealthier, and more likely to live in urban settings compared to women who deliver at home [6, 7].

Between 2000 and 2010*,* fifteen studies in Bangladesh, India, and Nepal collected data on self-reported obstetric complications and found that between 12% and 75% of women reported at least one complication in their most recent pregnancy [7–21]. These studies were limited by long recall periods, ranging from six months to five years, and retrospective designs [7–21]. Research from low-income settings suggests that biological factors are associated with obstetric complications [22, 23]. Studies show a U-shaped relationship between women’s age and risk of complications, with women less than 18 years and women older than 35 years at increased risk compared to women between 18 to 35 years [11, 24–26]. Nulliparous women are at increased risk of complications, particularly obstructed labor [24, 27, 28]. Adverse obstetric history, characterized by previous stillbirth or miscarriage, appears to increase risk of complications [24, 29–31]. Maternal malnutrition is associated with increased risk of obstetric complications and maternal deaths [32–34].

Beyond biological risk factors, socioeconomic and psychosocial factors are associated with obstetric complications [35, 36]. Studies in South Asia have linked poverty with adverse maternal health outcomes, possibly mediated by maternal illiteracy, lack of health information, and limited decision-making regarding reproductive health [36–41]. When pregnancies are wanted by both partners, data suggests that families are more likely to engage in optimal care-seeking behaviors [22, 23, 42, 43].

Experts recognize the need for high-quality, population-based data on obstetric complications [44, 45]. In this analysis we seek to explore the association of biological, socioeconomic, and psychosocial factors with reported obstetric complications, by type of complication, using data on symptoms assessed with minimal recall bias from a community setting in rural Bangladesh. We aim to identify opportunities to decrease the maternal morbidity burden in rural Bangladesh.

## Methods

### Context of parent trial

Conducted in northwest rural Bangladesh between 2007 and 2011, the JiVitA-3 community-randomized controlled trial enrolled 44,567 pregnant women to assess the effect of daily antenatal supplementation with multiple micronutrients, compared to iron-folic acid, on six-month infant mortality (Clintrials.gov #NCT00860470) [46]. The JiVitA-3 study area comprised 435 square kilometers of rural northwest Bangladesh, including 19 unions of Gaibandha and Rangpur Districts in Rangpur Division [47]. The study area was selected as representative of typical rural populations in Bangladesh based on population density (~1000 people per square kilometer), rural, agrarian characteristics (villages surrounded by rice fields, linked by unpaved roads), and economic and public health indicators [47]. As of 2010, Gaibandha District had a population of 2.3 million people, 51% of which was female and 49% male [48]. Literacy among females was reported at 63% [48].

Women were eligible for the parent trial if they could become pregnant (were of reproductive age 13–45 years, married and living with their husbands, not sterilized or menopausal, and whose husbands were not sterilized) [47]. These criteria have been used in trials in this same study area to define women at risk of becoming pregnant [49, 50]. At the outset, a census of all households within the study area was used to generate a comprehensive list of non-pregnant married women meeting above criteria. Eligible women were visited by field workers every five weeks to administer pregnancy tests for missed menses. Women who were identified as pregnant based on urine tests were asked for consent to enroll in the parent trial.

Enrolled women were visited weekly by trained female workers to ascertain pregnancy outcomes (live births, stillbirths, induced abortions, or miscarriages). Those who had a live birth or a stillbirth were administered a birth assessment form two weeks after delivery (standard deviation: 6 days) and asked whether they experienced a series of complications during labor and delivery. All women with a known pregnancy outcome were interviewed one month following the end of their pregnancy and asked whether they had experienced a series of symptoms in the four weeks before, during, or after the pregnancy outcome. Women were administered structured modules using local terms, pretested for comprehension in this setting, to assess symptoms. All women with pregnancy outcomes were asked: “At any time during pregnancy, delivery, or in the first four weeks after delivery, were you so sick that you believe you nearly died?” Data from these interviews was used to define the morbidity categories.

### Definitions of morbidity categories

Definitions of morbidity categories were modified from the WHO Integrated Management of Pregnancy and Childbirth (IMPAC) guidelines and previous studies on self-reported maternal morbidity in South Asia [51–54]. In Table 1, we show definitions for each complication category by type of pregnancy outcome. We focused on the major obstetric complications (eclampsia, sepsis, hemorrhage, and obstructed labor) as recommended in the IMPAC guidelines. Definitions with the highest ranges of sensitivity and specificity (based on validation studies conducted in Indonesia, the Philippines, and Ghana) were used for each complication category [55–57]. We included complications occurring during periods of high risk (the intrapartum period for women with births and the week following pregnancy outcomes for women with induced abortions) due to higher validity values for symptoms assessed in these time periods [55–57].

**Table 1:** **Operational definitions of obstetric complication and near misses by type of pregnancy outcome**

Category	Obstetric complication	Near miss
*Live Births or stillbirths - Complications during delivery*		
**Postpartum hemorrhage**	Report of profuse bleeding OR Retained placenta	Report of nearly dying + Profuse bleeding
	Sensitivity: 63-84%, Specificity: 89-97%	
**Eclampsia**	Convulsions (excluding epilepsy) and NO high fever	Report of nearly dying + Convulsions (excluding epilepsy) and NO high fever
	Sensitivity: 73-99%, Specificity: 86-99%	
**Sepsis**	High fever + foul-smelling vaginal discharge OR high fever + lower abdominal pain	Report of nearly dying + High fever
	Sensitivity: 69-74%, Specificity: 95-98%	
**Obstructed Labor**	(Report of baby stuck + use of saline drip with injection) OR (report of baby stuck + injection only) OR labor pains >24 hours	Report of nearly dying + (Report of baby stuck + use of saline drip with injection) OR (report of baby stuck + injection only) OR labor pains >24 hours
	Sensitivity: 56-70%, Specificity: 99-100%	
*Induced abortions or miscarriages*		
**Category**	**Obstetric complication**	**Near miss**
**Hemorrhage**	Report of vaginal bleeding (other than spotting) heavier than a normal period in 72 hours following pregnancy outcome	Report of nearly dying + Profuse bleeding
**Sepsis**	High fever + foul-smelling vaginal discharge OR high fever + lower abdominal pain in 7 days after pregnancy outcome	Report of nearly dying + High fever

Caption: These definitions are based on WHO’s Integrated Management of Pregnancy and Childbirth Guidelines [51]. The sensitivity and specificity values come from studies conducted in Indonesia, Philippines, and Ghana [55–57].

The eclampsia category consisted of report of convulsions (not related to epilepsy) in the absence of high fever, while sepsis was defined as high fever along with foul-smelling vaginal discharge or lower abdominal pain. Report of the baby having been stuck or labor pains greater than 24 hours, along with the use of saline with injection or injection only, was used to define obstructed labor. Women who reported profuse bleeding at birth or retained placenta were categorized as having had postpartum hemorrhage. For women with induced abortions or miscarriages, those who reported vaginal bleeding (other than blood spotting) heavier than a normal period in the three days after a pregnancy outcome were classified with hemorrhage (Table 1). Although the validity of definitions for abortive outcomes was not available from previous studies, we included IMPAC criteria (e.g. bleeding heavier than a normal period) to help distinguish abnormal bleeding that may result from induced abortion [51].

Women who reported that felt that they nearly died anytime during pregnancy, delivery, or 30 days after their pregnancy outcome as well as symptoms of eclampsia, hemorrhage, sepsis, or obstructed labor were considered “near misses.” Sensitivity and specificity values for the near miss category were not available from prior validation studies. Since IMPAC guidelines for obstetric complications exclude injuries [51], women who reported physical injuries, including those sustained from accidents, during pregnancy, delivery, or in the four weeks after their pregnancy outcome were excluded.

Women not meeting the criteria for obstetric complications or near misses as shown in Table 1 were categorized in the non-complicated pregnancy category. If women reported symptoms consistent with more than one morbidity, they were included in multiple groups. While the terms “obstetric complication” and “near miss” are defined by the criteria described above and shown in Table 1, the use of the term “maternal morbidity” in this analysis refers broadly to any illness symptom reported during pregnancy, delivery, or the 30 days following a pregnancy outcome.

### Definitions of independent variables

We focused on biological, socioeconomic, and psychosocial factors associated with obstetric complications and/or near misses in the literature. Upon enrollment in the parent trial, women’s socioeconomic characteristics were assessed, including ownership of household assets such as the number of bicycles and construction of homes. To measure socioeconomic status, we used an asset-based wealth index constructed using principal component analysis of household assets and construction materials, as described elsewhere [58]. Enrolled women were also interviewed about their literacy, defined as the ability to read or write a letter in Bengali (the local language).

At enrollment, demographic data such as number of previous pregnancies and the outcome of these pregnancies was also collected. This data was used to define adverse obstetric history as report of an adverse outcome (stillbirth or miscarriage) during the previous pregnancy. Women’s age was self-reported at enrollment. Parity was defined as the number of births a woman reported. Interviewers trained in obtaining anthropometric measurements measured the mid-upper arm circumference (MUAC) of women thrice, with the median value used for analysis. We included standard cutoffs used in the literature to distinguish women at higher thresholds of risk for complications due to age (<18 years and >35 years), parity (nulliparous), and wasting malnutrition in pregnant women (MUAC less than 21.5 cm) [25, 32, 44–50, 59, 60].

Upon enrollment in the parent trial, women were also asked whether they wanted the index pregnancy and whether their husbands had wanted the pregnancy. A ranking of couples’ pregnancy wantedness was created, ranging from both partners wanting the pregnancy, only one partner wanting the pregnancy, and neither partner wanting the pregnancy.

### Data analysis

To explore the data, we tabulated frequency distributions for categorical variables and used Chi-squared tests to assess differences between the multiple morbidity categories. Multinomial logistic regression was used to obtain relative risk ratios (RRR) and 95% confidence intervals (CI) to estimate the relationships of independent variables with three morbidity categories defined as those with non-complicated pregnancies (referent category) and those who had obstetric complications and near misses, respectively. Robust cluster estimates were generated to account for cluster-randomization in the parent trial using the Huber-White sandwich estimator [61]. We retained variables strongly associated with development of obstetric complications in the literature (women’s age, obstetric history) as well as potential confounders (year and season of pregnancy outcome) in the base model. Selection procedures based on Akaike Information Criteria values were used to identify optimal regression models. Model fit was assessed by comparing the log likelihoods of the fitted model to the intercept-only model.

Sub-analyses by the type of complication reported (hemorrhage, eclampsia, sepsis, or obstructed labor), was done using logistic regression adjusted for year and season of pregnancy outcome, as recorded in the parent trial. All data analysis was performed in Stata 11 [62].

The JiVitA-3 trial and all interim follow-up studies received ethical approval from the Bangladesh Medical Research Council (BMRC Reference Number 458) and the Johns Hopkins School of Public Health Institutional Review Board (IRB 00000570). The JiVitA-3 trial is registered under ClinicalTrials.gov NCT00860470.

## Results

### Characteristics of study sample

**Figure 1:**
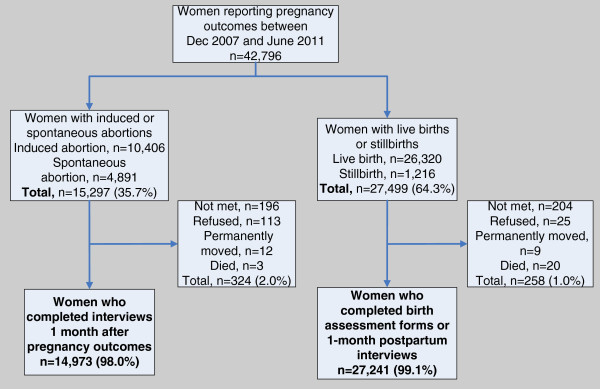
**Analytic cohort of 42,214 married women of reproductive age in rural northwest Bangladesh, by type of pregnancy outcome.** Figure 1 shows the analytic cohort of married women of reproductive age completing birth assessment interviews or one-month postpartum interviews (n=42,214) in rural northwest Bangladesh between December 2007 and June 2011, by type of pregnancy outcome.

As a subset of the pregnant women enrolled in the JiVitA-3 parent trial, this analysis drew from the 42,796 women who reported pregnancy outcomes between December 2007 and June 2011 (Figure 1). Approximately 61% of women had live births (n = 26,320) and 3% had stillbirths (n = 1,216), while 25% (n = 10,406) of women had induced abortions and 11% (n = 4,891) had miscarriages. The 42,214 women who completed maternal morbidity interviews (99% of the women with pregnancy outcomes) were considered the analytic cohort for this paper.

Using the definitions in Table 1, 25% (n = 10,380) of women were classified as having obstetric complications, 2% (n = 1,004) with near misses, and 73% (n = 30,830) with non-complicated pregnancies. Hemorrhage was identified as the leading complication, reported by 12% (n = 5,232) of all pregnant women, followed by sepsis (8%, n = 3,259). Of the 27,241 women with live births or stillbirths, 11% (n = 2,950) reported obstructed labor and 1% (n = 328) reported eclampsia.

Table 2 describes the distribution of biological, socioeconomic, and psychosocial characteristics by morbidity categories. The mean age of women at the time of pregnancy was 24.0 years (standard deviation: 6.5 years), with 27% of women reporting no previous births and 73% having had at least one previous birth. Approximately 57% of women were literate and 42% were employed. Women had a mean four years of schooling (standard deviation: 3.8 years), while their husbands had three years (standard deviation: 2.9 years) (data not shown). Compared to women with non-complicated pregnancies, a higher proportion of women reporting obstetric complications were classified as nulliparous (p < 0.001), with adverse obstetric history (p < 0.001), and with neither partner wanting the pregnancy (p < 0.001), among other factors (Table 2).

**Table 2:** **Distribution of socioeconomic, biological, and psychosocial characteristics among 42,214 women in rural northwest Bangladesh (2007–2011)**

Characteristics	Non-complicated pregnancy	Obstetric complication	Near miss	Total
	n = 30,830	n = 10,380	n = 1,004	n = 42,214
	%	%	%	% (n)
*Biological factors*				
Women’s age***				
<18 years	13.3	19.5	15.3	15.2 (6,417)
18-35 years	80.9	70.5	80.3	78.6 (33,180)
>35 years	5.7	9.9	7.1	6.0 (2,592)
Parity***				
Nulliparity	24.6	34.2	34.4	27.2 (11,479)
1-2 births	49.8	36.7	36.8	46.3 (19,556)
>2 births	25.5	29.0	28.8	26.4 (11,172)
Obstetric history***				
No previous stillbirth or miscarriage	77.3	71.9	74.3	77.4 (36,197)
Stillbirth or miscarriage	22.7	28.0	25.6	22.5 (6,007)
Mid-upper arm circumference*				
< 21.5 cm	33.4	37.6	34.7	33.8 (14,226)
≥ 21.5 cm	66.4	62.3	65.2	65.8 (27,819)
*Socioeconomic factors*				
***Household wealth index***				
Lowest quartile	25.3	25.5	25.7	25.7 (10,836)
2^nd^ quartile	25.6	25.5	24.8	25.3 (10,662)
3^rd^ quartile	24.7	25.3	24.3	24.8 (10,473)
Highest quartile	24.3	23.5	25.2	24.1 (10,186)
Women’s literacy*				
Illiterate	45.2	39.8	44.5	43.2 (18,241)
Literate	54.7	60.0	55.5	56.7 (23,913)
*Psychosocial factors*				
Couples’ pregnancy wantedness ranking***				
Both partners wanted pregnancy	43.0	20.1	36.7	39.2 (16,616)
Only one partner wanted pregnancy	11.1	17.4	12.0	10.8 (4,512)
Neither partner wanted pregnancy	45.1	61.9	50.6	49.3 (20,805)

*p-value <0.05 of overall difference between the three groups using Chi-squared tests.

***p-value < 0.001 of overall difference between the three groups using Chi-squared tests.

Table 2 shows the distribution of socioeconomic, biological, and psychosocial characteristics among 42,214 women reporting obstetric complications, near misses, or non-complicated pregnancies in rural northwest Bangladesh between December 2007 and June 2011. Data was collected up to June 2011. A total of 619 women had missing data on characteristics of interest; 25 women were missing data on age, 7 had missing data on parity, 19 women had missing data on obstetric history, 169 had missing data on mid-upper arm circumference, 57 were missing data on household wealth index, 61 women had missing data on literacy, and 281 were missing data on pregnancy wantedness.

Among the 27,241 women with live births or stillbirths, 85% (n = 23,155) gave birth in their home or a relative’s home, 14% gave birth in health facilities, and 1% gave birth in the home of a health provider trained to perform deliveries (nurse or family welfare visitors) (Table 3). Fifteen percent of women had skilled attendants at birth (defined as doctors, nurses, or other government health workers trained to perform deliveries [5]), while 39% of women were attended by friends, relatives, or neighbors only, 29% by untrained traditional birth attendants (TBA, local term: “*dai*”), and 17% by trained TBAs. Six percent of women were attended by both friends/relatives/neighbors and untrained TBAs. Among the 26,283 women with live births, 27% reported attending at least one antenatal visit, with 73% of women reporting no antenatal care (Table 3).

**Table 3:** **Delivery characteristics among 27,241 women with live births or stillbirths in rural northwest Bangladesh (2007–2011)**

Characteristics	% (n)
*Place of delivery (among 27,241 women with live births or stillbirths)*	
Home	86% (23,427)
Health facility	14% (3,814)
*Skilled attendance at birth* ^a^ *(among 27,241 women with live births or stillbirths)*	
Doctor, nurse, or family welfare visitor	15% (4,086)
Relatives, family, or neighbors only	45% (12,389)
Untrained TBA	29% (7,900)
Trained TBA	17% (4,710)
*Antenatal care visits* ^*b*^ *(among 26,283 women with live births)*	
At least one visit	27% (7,096)
No visits	73% (19,187)

*Abbreviations:* TBA = traditional birth attendant.

^a^Multiple attendants could be listed. 948 women with untrained TBAs also reported relatives, family members, or neighbors present. 896 women with trained TBAs also reported untrained TBAs, relatives, family, or neighbors present.

^b^Data on ANC visits was available for 26,283 women with at least one live birth.

### Bivariate and multivariable analyses

From the analytic cohort of 42,214 women, 41,660 (99%) had data on all independent variables of interest and were included in regression models. We first analyzed bivariate associations of independent variables with morbidity categories (Table 4). In this unadjusted analysis, biological risk factors including women’s age (less than 18 years: RRR :1.28 95% CI: 1.11-1.47; greater than 35 years: RRR 1.24 95% CI 1.10-1.39), nulliparity (RRR 1.36 95% CI 1.15-1.58), adverse obstetric history (RRR 1.17 95% CI 1.05-1.30), and MUAC < 21.5 cm (RRR 1.14 95% CI 1.02-1.26) appeared to increase the risk of reported obstetric complications (Table 3). Lack of pregnancy wantedness among couples (only one partner wanting pregnancy: RRR 1.12 95% CI 1.05-1.20; neither partner wanting pregnancy: RRR 1.41 95% CI 1.30-1.52) also increased the risk of reported obstetric complications. Literacy was positively and significantly associated with reported obstetric complications in bivariate analysis (RRR 1.08 95% CI 1.01-1.14), while household wealth index was not significant (Table 4).

**Table 4:** **Unadjusted relative risk ratios of factors associated with obstetric complications among 41,660 rural Bangladeshi women**

	Obstetric complications (n = 10,217)		Near misses (n = 993)	
Independent variables	Relative risk ratio (RRR)	95% confidence interval	Relative risk ratio (RRR)	95% confidence interval
**Biological factors**				
*Age 18–35 years (ref)*				
Age < 18 years	1.28***	(1.11, 1.47)	1.21	(0.97, 1.47)
Age >35 years	1.24***	(1.10, 1.39)	1.25	(0.98, 1.53)
*Parity 1–2 (ref)*				
Nulliparity	1.36***	(1.15, 1.58)	1.29***	(1.11, 1.46)
Parity > 2	1.13	(0.99, 1.27)	1.16	(0.98, 1.31)
*No previous stillbirth or miscarriage (ref)*				
Adverse obstetric history	1.17***	(1.05, 1.30)	1.15	(0.97, 1.32)
*MUAC ≥ 21.5 cm (ref)*				
MUAC < 21.5 cm	1.14*	(1.02, 1.26)	1.04	(0.91, 1.17)
**Socioeconomic factors**				
*Wealth index – Lowest quartile (ref)*				
Wealth index - 2^nd^ quartile	1.00	(0.87, 1.13)	0.99	(0.89, 1.10)
Wealth index - 3^rd^ quartile	1.03	(0.90, 1.16)	0.99	(0.90, 1.09)
Wealth index - Highest quartile	0.99	(0.89, 1.10)	1.02	(0.89, 1.14)
*Maternal illiteracy (ref)*				
Maternal literacy	1.08*	(1.01, 1.14)	0.99	(0.81, 1.17)
**Psychosocial factors**				
*Both partners wanted pregnancy (ref)*				
Only one partner wanted pregnancy	1.12**	(1.05, 1.20)	1.05	(0.88, 1.23)
Neither partner wanted pregnancy	1.41***	(1.30, 1.52)	1.07	(0.89, 1.23)

*p-value <0.05.

**p-value <0.01.

***p-value <0.001.

Table 4 shows unadjusted bivariate associations from multinomial logistic regression of socioeconomic, biological, and psychosocial factors with reported obstetric complications and near misses (compared to 30,450 non-complicated pregnancies) among 41,660 married women of reproductive age with data on all independent variables in rural northwest Bangladesh from December 2007 to June 2011.

In the adjusted multinomial regression model (Table 5), women’s age less than 18 years (RRR 1.26 95% CI 1.14-1.39) and greater than 35 years (RRR 1.23 95% CI 1.09-1.38), nulliparity (RRR 1.16 95% CI 1.02-1.29), obstetric history of miscarriage or stillbirth (RRR 1.15 95% CI 1.07-1.22), and neither partner wanting the pregnancy (RRR 1.33 95% CI 1.20-1.46) significantly increased the risk of reported obstetric complications. Nulliparity remained the only factor that was significantly associated with reported near miss (RRR 1.20 95% CI 1.06-1.34) in the adjusted analysis. The association of mid-upper arm circumference with obstetric complications became non-significant (RRR 1.07 95% CI 0.99-1.14) in the adjusted model, as did maternal literacy (RRR 1.01 95% CI 0.92-1.12) (Table 5).

**Table 5:** **Adjusted relative risk ratios of factors associated with obstetric complications among 41,660 rural Bangladeshi women**

	Obstetric complications (n = 10,217)		Near misses (n = 993)	
Independent variables	Relative risk ratio (RRR)	95% confidence interval	Relative risk ratio (RRR)	95% confidence interval
**Biological factors**				
*Age 18–35 years (ref)*				
Age <18 years	1.26***	(1.14, 1.39)	1.16	(0.98, 1.34)
Age >35 years	1.23***	(1.09, 1.38)	1.14	(0.94, 1.35)
*Parity 1–2 (ref)*				
Nulliparity	1.16**	(1.02, 1.29)	1.20**	(1.06, 1.34)
Parity > 2	1.10	(0.99, 1.22)	1.11	(0.96, 1.25)
*No previous stillbirth or miscarriage (ref)*				
Adverse obstetric history	1.15***	(1.07, 1.22)	1.13	(0.98, 1.29)
*MUAC ≥ 21.5 cm (ref)*				
MUAC < 21.5 cm	1.07	(0.99, 1.14)	1.00	(0.89, 1.11)
**Socioeconomic factors**				
*Wealth index – Lowest quartile (ref)*				
Wealth index - 2^nd^ quartile	1.00	(0.90, 1.11)	1.00	(0.91, 1.10)
Wealth index - 3^rd^ quartile	1.01	(0.89, 1.12)	1.01	(0.90, 1.13)
Wealth index - Highest quartile	1.00	(0.92, 1.09)	1.01	(0.89, 1.12)
*Maternal illiteracy (ref)*				
Maternal literacy	1.01	(0.92, 1.12)	1.00	(0.90, 1.11)
**Psychosocial factors**				
*Both partners wanted pregnancy (ref)*				
Only one partner wanted pregnancy	1.07	(0.98, 1.16)	1.01	(0.87, 1.16)
Neither partner wanted pregnancy	1.33***	(1.20, 1.46)	1.03	(0.87, 1.19)

**p-value <0.01.

***p-value <0.001.

Table 5 shows adjusted relative risk ratios from multivariable multinomial logistic regression assessing the association of socioeconomic, biological, and psychosocial factors with reported obstetric complications and near misses (compared to 30,450 non-complicated pregnancies) among 41,660 married women of reproductive age with data on all independent variables in rural northwest Bangladesh from December 2007 to June 2011. Results are adjusted by year and season of pregnancy outcome.

### Sub-analyses

For sub-analyis, we tested the association of independent variables with reported obstetric complications or near misses, stratified by type of complication. The biological factors of women’s age, obstetric history, and parity appeared to be significantly associated with all types of complications (Table 6). For eclampsia, these biological factors were the only factors that were significantly associated with reported obstetric complications. Mid-upper arm circumference less than 21.5 cm significantly increased the risk of reported sepsis (OR 1.16 95% CI 1.04-1.18) and hemorrhage (OR 1.12 95% CI 1.03-1.20), while neither partner wanting the pregnancy increased the risk of reported sepsis, obstructed labor, and hemorrhage (Table 6). Among women with live births, receipt of any antenatal care was protective against obstetric complications (OR 0.88 95% CI 0.82-0.94) (Table 7), whereas delivery in health facility was associated with obstetric complications among women with live births or stillbirths (OR 0.90 95% CI 0.89-0.99) (Table 8).

**Table 6:** **Adjusted odds ratios of factors associated with obstetric complications or near misses, by type of complication**

	Reported obstetric complications or near misses			
Independent variables	Eclampsia	Hemorrhage	Sepsis	Obstructed labor
	(n = 324)	(n = 4,945)	(n = 3,120)	(n = 2,847)
	Odds ratio	Odds ratio	Odds ratio	Odds ratio
**Biological factors**				
*Age 18–35 years (ref)*				
Age <18 years	1.35***	1.20***	1.18***	1.27***
Age >35 years	1.34***	1.27***	1.19*	1.21***
*Parity 1–2 (ref)*				
Nulliparity	1.13**	1.10**	1.14**	1.30***
Parity >2	1.12	1.09	1.05	1.10
*No previous stillbirth or miscarriage (ref)*				
Adverse obstetric history	1.18***	1.06**	1.12**	1.14***
*MUAC ≥ 21.5 cm (ref)*				
MUAC < 21.5 cm	1.04	1.12*	1.16*	1.05
**Socioeconomic factors**				
*Wealth index – Lowest quartile (ref)*				
Wealth index - 2^nd^ quartile	1.00	1.01	1.02	1.00
Wealth index - 3^rd^ quartile	1.01	1.02	1.00	1.00
Wealth index - Highest quartile	1.00	1.03	1.05	1.02
*Maternal illiteracy (ref)*				
Maternal literacy	1.01	1.01	1.04	1.03
**Psychosocial factors**				
*Both partners wanted pregnancy (ref)*				
Only one partner wanted pregnancy	1.02	1.06	1.08	1.07
Neither partner wanted pregnancy	1.02	1.40***	1.37***	1.28***

*p-value <0.05.

**p-value <0.01.

***p-value <0.001.

**Table 7:** **Adjusted odds ratios of factors associated with obstetric complications or near misses among women with live births**

Independent variables	Live births (n = 25,687)
**Biological factors**	
*Age 18–35 years (ref)*	
Age <18 years	1.16***
Age >35 years	1.20***
*Parity 1–2 (ref)*	
Nulliparity	1.19***
Parity > 2	1.12
*No previous stillbirth or miscarriage (ref)*	
Adverse obstetric history	1.14***
*MUAC ≥ 21.5 cm (ref)*	
MUAC < 21.5 cm	1.08
**Socioeconomic factors**	
*Wealth index – Lowest quartile (ref)*	
Wealth index - 2^nd^ quartile	1.00
Wealth index - 3^rd^ quartile	1.01
Wealth index - Highest quartile	1.00
*Maternal illiteracy (ref)*	
Maternal literacy	1.04
**Psychosocial factors**	
*Both partners wanted pregnancy (ref)*	
Only one partner wanted pregnancy	1.00
Neither partner wanted pregnancy	1.16***
**Care during pregnancy**	
*Did not attend any antenatal visits (ref)*	
Attended at least one antenatal visit	0.88**

Results are adjusted by year and season of pregnancy outcome. ANC data available on women with live births only.

**p-value <0.01.

***p-value <0.001.

**Table 8:** **Adjusted odds ratios of factors associated with obstetric complications or near misses among women with live births or stillbirths**

Independent variables	Live births or stillbirths (n = 26,903)
**Biological factors**	
*Age 18–35 years (ref)*	
Age <18 years	1.19***
Age >35 years	1.25***
*Parity 1–2 (ref)*	
Nulliparity	1.12**
Parity > 2	1.10
*No previous stillbirth or miscarriage (ref)*	
Adverse obstetric history	1.17***
*MUAC ≥ 21.5 cm (ref)*	
MUAC < 21.5 cm	1.05
**Socioeconomic factors**	
*Wealth index – Lowest quartile (ref)*	
Wealth index - 2^nd^ quartile	1.02
Wealth index - 3^rd^ quartile	0.99
Wealth index - Highest quartile	1.00
*Maternal illiteracy (ref)*	
Maternal literacy	1.06
**Psychosocial factors**	
*Both partners wanted pregnancy (ref)*	
Only one partner wanted pregnancy	1.03
Neither partner wanted pregnancy	1.19***
**Place of delivery**	
*Home (ref)*	
Health facility	0.90*

Results are adjusted by year and season of pregnancy outcome. ANC data available on women with live births only.

*p-value <0.05.

**p-value <0.01.

***p-value <0.001.

## Discussion

### Main findings

Using population-based data on symptoms assessed with minimal recall bias from a rural setting of Bangladesh, our analysis shows that a quarter of the women reported obstetric complications, with two percent reporting near misses. Biological risk factors and lack of pregnancy wantedness were associated with increased risk of obstetric complications, while parity was the only factor that was significantly associated with reported near misses. MUAC less than 21.5 cm was a risk factor for reported hemorrhage or sepsis.

### Interpretation

The estimates in our study are consistent with the proportions of self-reported complications found in other studies from South Asia. Population-based studies published since 2005 have indicated that between 15% and 38% of women experienced an obstetric complication during their most recent pregnancy [13, 14, 21, 52, 53]. The prevalence in our study (25% of women of reproductive age with recent pregnancies reporting obstetric complications) falls within the middle range of these estimates. Our numbers are closest to the proportions estimated in West Bengal and in Uttar Pradesh, India where 23% of women with pregnancies in the four years prior to survey (n = 3,111) and 21% of women with pregnancies in the year prior to survey (n = 410), respectively, were considered to have intrapartum obstetric complications [11, 12]. This similarity may arise from the common, though not identical, definitions used for obstetric complications.

For individual complications, the proportion of women reporting hemorrhage in our study (12%) falls within the range of the 5% to 24% of surveyed women of reproductive age who reported “excessive bleeding” in studies conducted in South Asia [7, 10–13, 16–18, 20, 53]. Our estimates of sepsis and eclampsia (8% and 1% of women, respectively) lie in the lower range of the proportions reported in previous studies [11, 12, 15, 17, 24, 54]. The lower proportions in our study could arise from our use of more exclusive definitions as well as shorter recall periods.

The prevalence of self-reported near misses recorded in this study (2%) is similar to the 2% of women admitted to intensive care units or delivery wards considered to have near misses in hospital-based studies in Nepal [63], Morocco [64], and Brazil [65, 66]. The prevalence of obstetric complications in our study (25%) is similar to 23% of women considered to have obstetric complications in a hospital-based study in Matlab, Bangladesh, a demographic surveillance site where rates of institutional deliver are higher (50%) than the national average (29%) for institutional delivery [67]. Although our definitions were not based on clinical criteria, we may have captured severe morbidity since we restricted definitions to structured symptoms shown to have high validity in previous studies [55–57].

In our analysis, biological factors appeared to increase the risk of all reported obstetric complications. Research from the JiVitA study area has suggested that adolescents who become pregnant may be unable to meet the increased nutritional demands of pregnancy and lactation [68], which could increase risk for obstetric complications. In this setting, child marriage is strongly tied with early childbearing, with girls who marry early also likely to drop out of school and have low decision-making power in their families [44, 69]. Biological factors such as adverse obstetric history and nulliparity, which have been associated with risk of complications in previous studies, were all significant risk factors in this analysis [11, 24–26]. Women’s age above 35 years has also been associated with elevated risk for pregnancy-related mortality, as shown in a prospective community-based study on 25,580 pregnancies in rural Nepal conducted by Christian et al. [32]. The consistency of biological risk factors in this analysis with previous studies may suggest validity of the assessed morbidities to detect obstetric complications.

Maternal mid-upper arm circumference less than 21.5 cm, a cutoff that may be indicative of wasting malnutrition in pregnant women [32], appeared to increase the risk of reported hemorrhage or sepsis. Christian et al. found that rural Nepalese women with MUAC less than 21.5 cm had a 1.5 increased risk of pregnancy-related mortality [32]. Studies have shown associations between wasting malnutrition and weakened immune system response to infection [68, 70], as well as associations between acute malnutrition and anemia in pregnancy [71]. Data from rural northwest Bangladesh suggests a high prevalence of anemia in early pregnancy, with 26% of 42,896 pregnant women between 2001 and 2007 estimated to be anemic [60]. In this analysis, a high proportion of pregnant women (33%) were estimated to have MUAC below 21.5 cm.

Women’s report of neither partner wanting the index pregnancy was associated with increased risk of reported obstetric complications. Studies have suggested that pregnancy wantedness may indicate the importance placed on the pregnancy and the care and attention given to women throughout pregnancy [22, 23]. Lack of pregnancy wantedness has been associated with decreased attendance at antenatal visits [22, 23]. Only 39% of women in this study indicated that both partners wanted the pregnancy, suggesting substantial unmet need for contraception in this population.

In this analysis, a quarter of the women reported having an induced abortion. While abortions performed under safe conditions incur low risk of complications, unsafe abortion can lead to complications such as hemorrhage, sepsis and chronic morbidity [72]. In Bangladesh, menstrual regulation, the vacuum extraction of intrauterine content to stimulate menstruation and terminate pregnancy [73], is a legal procedure. While menstrual regulation has been credited for a marked decline in abortion-related deaths over the last few decades, abortions induced by untrained TBAs remain a major cause of morbidity in developing countries [72]. A previous study in rural Bangladesh showed that women reported seeking crude methods of pregnancy termination when unable to pay for safer medical abortions [74]. Safe abortion procedures coupled with post-abortion care and follow-up referral, as indicated, need to be advocated and made more accessible to women wishing to terminate their pregnancies in rural areas. Future studies may compare maternal outcomes for women with safe versus unsafe methods of induced abortion.

Although antenatal care attendance is widely recommended for identification of potential risk factors, encouragement of health behaviors, and planning for complications that may arise [29], less than one-third of women (27%) who had live births in our study attended any antenatal visits. Increasing attendance at ANC visits could increase awareness of potential complications among pregnant women as well as encourage emergency preparedness and planning.

At the near-miss level of morbidity, socioeconomic and psychosocial factors were non-significant compared to the biological risk factor of parity. Using data from hospital-based studies, authors have suggested that socioeconomic factors may cease to be important for near misses due to their severity [75, 76]. For women who died from pregnancy-related causes, research from Nepal also indicates that socioeconomic factors did not significantly impact the risk of death, possibly due to the severity of these conditions and the relatively uniform distribution of socioeconomic status in rural areas [32].

### Strengths and limitations

This analysis used prospective data on structured symptoms comprising obstetric complications and near misses, assessed within two to eight weeks to minimize recall bias. Detailed morbidity modules facilitated the exploration of results by complication (hemorrhage, obstructed labor, sepsis, and eclampsia) and by type of pregnancy outcome. Data on socioeconomic, biological, and psychosocial factors of interest were collected and available for analysis. The large sample size provided sufficient power for main analyses and sub-analysis.

Since the data in the parent trial were not exclusively collected to determine risk factors for reported complications, we were unable to make direct causal inferences. However, the presented associations exhibited plausible relationships and coherence with previous studies. Since our analyses were based on women’s report of complications rather than clinically confirmed complications, these definitions may have low validity for estimates of prevalence of clinically relevant conditions. However, we applied definitions of obstetric complications recommended by WHO IMPAC guidelines for community settings in order to reduce potential misclassification. Although we were unable to validate the specific definitions used in this study for this population, we have provided sensitivity and specificity values, when available, for similar definitions used in resource-poor settings.

## Conclusions

Using population-based data on symptoms assessed with minimal recall bias from a rural setting of Bangladesh, this analysis shows that a quarter of women reported obstetric complications, with biological factors and lack of pregnancy wantedness increasing the risk of complications. Additional research from rural communities on risk factors for complications, assessed with minimal recall bias, may help to illuminate the pathways by which risk factors may affect development of obstetric complications. Future research on obstetric complications in rural communities should consider validating self-reported complications with clinical classifications.

Policies to reduce early marriage and unmet need for contraception may facilitate behavior modification of risk factors such as early childbearing. Increasing antenatal visit coverage could facilitate identification of biological risk factors, including assessment of undernutrition, for obstetric complications as well as to promote preparedness for complications that may arise. While safe motherhood strategies are centered on improving utilization and access to facility delivery, continued focus is needed on addressing the significant burden of complications that still occur in the home across rural communities of South Asia.
